# Matrix-induced autologous chondrocyte implantation (mACI) versus autologous matrix-induced chondrogenesis (AMIC) for chondral defects of the knee: a systematic review

**DOI:** 10.1093/bmb/ldac004

**Published:** 2022-02-16

**Authors:** Filippo Migliorini, Jörg Eschweiler, Christian Götze, Arne Driessen, Markus Tingart, Nicola Maffulli

**Affiliations:** 1 Department of Orthopaedic and Trauma Surgery, RWTH University Hospital Aachen, Pauwellstr. 31, 52074 Aachen, Germany; 2 Department of Orthopaedic Surgery, Auguste-Viktoria Clinic, Ruhr University Bochum, Am Kokturkanal 2, 32545 Bad Oeynhausen, Germany; 3 Department of Medicine, Surgery and Dentistry, University of Salerno, Via S. Allende, 84081 Baronissi (SA), Italy; 4 Queen Mary University of London, Barts and the London School of Medicine and Dentistry, Centre for Sports and Exercise Medicine, Mile End Hospital, 275 Bancroft Road, London E1 4DG, UK; 5 School of Pharmacy and Bioengineering, Keele University Faculty of Medicine, Thornburrow Drive, Stoke on Trent, ST5 5BG, UK

**Keywords:** Knee, chondral defect, mACI, AMIC

## Abstract

**Introduction:**

Chondral defects of the knee are common and their treatment is challenging.

**Source of data:**

PubMed, Google scholar, Embase and Scopus databases.

**Areas of agreement:**

Both autologous matrix-induced chondrogenesis (AMIC) and membrane-induced autologous chondrocyte implantation (mACI) have been used to manage chondral defects of the knee.

**Areas of controversy:**

It is debated whether AMIC and mACI provide equivalent outcomes for the management of chondral defects in the knee at midterm follow-up. Despite the large number of clinical studies, the optimal treatment is still controversial.

**Growing points:**

To investigate whether AMIC provide superior outcomes than mACI at midterm follow-up.

**Areas timely for developing research:**

AMIC may provide better outcomes than mACI for chondral defects of the knee. Further studies are required to verify these results in a clinical setting.

## Introduction

Hyaline cartilage tissue is alymphatic and hypocellular, with low metabolic activity and limited regenerative capabilities.^1–3^ The healing process of chondrocytes often does not result in *restitutio ad integrum,* and residual chondral defects or a fibrotic scar are frequent.^4^^,^^5^ Focal chondral defects of the knee are debilitating, leading to marked decline in quality of life and, in athletes, a high chance of retirement from sport.^6^^,^^7^ Conservative strategies are often not adequate to manage focal chondral defects of the knee.^8^^,^^9^ Thus, surgical management is often required.^10^^,^^11^ Several different surgical strategies have been proposed to manage focal chondral defects of the knee.^12–14^ After its introduction, membrane-induced autologous chondrocyte implantation (mACI) has been broadly performed.^11^^,^^15^^,^^16^ In 2005, Behrens^17^ first described an enhanced microfractures technique, which quickly evolved into the autologous matrix-induced chondrogenesis (AMIC) procedure. Given its simplicity, AMIC quickly gained the favour of surgeons and patients.^18^ To the best of our knowledge, no previous study compared these two strategies in a clinical setting for chondral defect of the knee. AMIC was supposed to perform better than the mACI procedure; however, no consensus has been reached, and updated evidenced-based recommendations are required. Thus, a systematic review was conducted to investigate whether AMIC provides better outcomes than mACI for knee chondral defects at midterm follow-up. This study focused on patient-reported outcome measures (PROMs) and complication rates. We hypothesized that AMIC and mACI procedures provided equivalent clinical outcome.

## Method

### Search strategy

This systematic review followed the Preferred Reporting Items for Systematic Reviews and Meta-Analyses (PRISMA).^19^ The PICO algorithm was preliminarily stated:

- P (Problem): knee chondral defect;
- I (Intervention): chondral regeneration;
- C (Comparison): AMIC versus mACI;
- O (Outcomes): PROMs and complications.

### Data source and extraction

The literature search was conducted by two authors (Filippo Migliorini1 and Jörg Eschweiler) separately in January 2022. The following databases were accessed: PubMed, Google scholar, Embase and Scopus. The following keywords were used in combination: *chondral, cartilage, articular, damage, defect, injury, chondropathy, knee, pain, matrix-induced, autologous, chondrocyte, transplantation, implantation, mACI, AMIC, therapy, management, surgery, outcomes, hypertrophy, failure, revision, reoperation, recurrence.* The same authors independently screened the resulting articles from the search. The full-text of the articles of interest was accessed. A cross-reference of the bibliographies was also performed. Disagreements between the two authors were solved by a third author (Nicola Maffulli).

### Eligibility criteria

All the studies investigating the outcomes of AMIC and/or mACI for knee chondral defects were accessed. Given the authors language abilities, articles in English, Italian, French, Spanish and German were eligible. Levels I to IV of evidence studies, according to the Oxford Centre of Evidence-Based Medicine,^20^ were suitable. Only studies investigating a minimum of five patients were included. Abstracts, reviews, letters, opinion, editorials and registries were excluded. Biomechanics, animals or *in vitro* studies were not considered. Only studies that used a cell-free bioresorbable membrane were considered. Studies augmenting AMIC or mACI with less committed cells (e.g. bone marrow concentrate, mesenchymal stem cells) or grow factors were not considered. Studies involving patients with kissing lesions were not included, nor were those involving patients with end-stage osteoarthritis. Only studies that clearly stated the duration of the follow-up were eligible. Only studies which reported quantitative data with regards to the outcomes of interest were included in this study.

### Data extraction

Data extraction was conducted independently by two authors (Filippo Migliorini1 and Jörg Eschweiler). Generalities of the included studies (author and year, journal, study design) and patients demographic at baseline were collected (length of symptoms prior of treatment, number of procedures, mean body mass index (BMI) and age of the patients, length of the follow-up, gender, mean defect size). For each of the two techniques, the following data were retrieved: Visual Analogue Scale (VAS), Tegner Activity Scale,^21^ International Knee Documentation Committee (IKDC)^22^ and the Lysholm Knee Scoring Scale.^23^ Data regarding the following complications were also collected: rate of hypertrophy, failures, revision surgeries and total knee arthroplasty. The recurrence of symptomatic chondral defects which affect negatively the patient quality of life was considered as failure.

### Methodology quality assessment

The methodological quality assessment was accomplished by two independent authors (Filippo Migliorini1 and Jörg Eschweiler). The risk of bias graph tool of the Review Manager Software (The Nordic Cochrane Collaboration, Copenhagen) was used. The following risks of bias were evaluated: selection, detection, attrition, reporting and other sources of bias.

### Statistical analysis

The statistical analysis was performed with IBM SPSS Version 25. Continuous data were reported as mean difference (MD), while binary data were evaluated using the odd ratio (OR) effect measure. The confidence interval (CI) was set at 95% in all the comparisons. T-test and $\chi$^2^ were evaluated for continuous and binary data, respectively, with *P* < 0.05 considered statistically significant.

## Results

### Search result

**Fig. 1:**
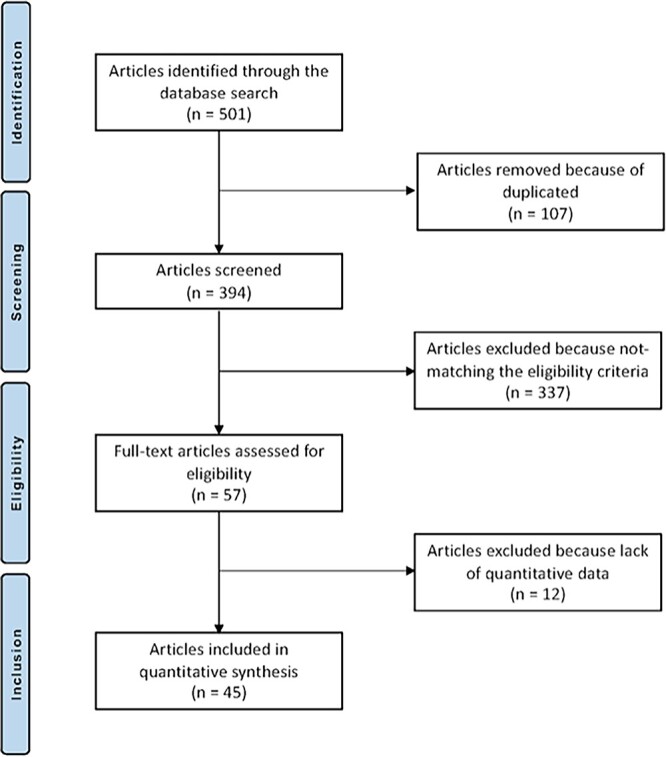
Flow chart of the literature search.

A total of 503 articles were initially obtained and 107 were excluded as they were duplicates. A further 349 articles were excluded because they did not match the inclusion criteria: not focused on mACI or AMIC (*N* = 225), not focusing on knee (*N* = 37), study design (*N* = 51), not reporting quantitative data under the outcomes of interest (*N* = 12), combined with other committed cells (*N* = 12), other (*N* = 8), language limitations (*N* = 3), not clearly stating the duration of the follow-up (*N* = 1). Finally, 47 articles were available for this study. The results of the literature search are shown in Figure 1.

### Methodological quality assessment

**Fig. 2:**
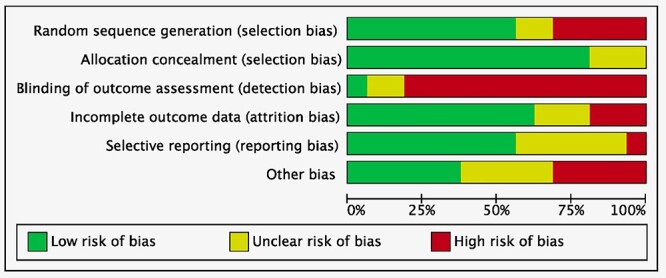
Methodological quality assessment.

As 27% (12 of 45) of the investigations were randomized clinical trials, and 20% (9 of 45) were retrospective studies, the risk of selection bias of random sequence generation was moderate. The overall risk of selection bias of allocation concealment was low. Given the overall lack of blinding, detection bias was moderate-high. The risk of attrition and reporting bias across all included studies was low, as was the risk of other bias. In conclusion, the risk of bias was moderate, attesting to this study acceptable methodological assessment (Fig. 2).

### Patient demographics

Data from 1667 procedures were retrieved; 36% (600 of 1667 patients) were women. The mean follow-up was 37.9 ± 21.7 months. The mean age of the patients was 34.7 ± 6.5, and the mean BMI 25.5 ± 1.6 kg/m^2^. The mean defect size was 3.9 ± 1.2 cm^2^. Generalities and demographics of the study are shown in Table 1.

Good comparability was found between the two groups at baseline (Table 2).

### Outcomes of interest

The AMIC group demonstrated greater values of IKDC (MD 7.7; *P* = 0.03) and Lysholm (MD 16.1; *P* = 0.02) scores. Similarity was found concerning the VAS (*P* = 0.5) and Tegner (*P* = 0.2) scores (Table 3).

### Complications

The AMIC group demonstrated lower rate of failures (OR 0.2; *P* = 0.04). Similarity was found concerning the rate of hypertrophy (*P* = 0.05), knee arthroplasty (*P* = 0.4) and revision surgery (*P* = 0.07) (Table 4).

**Table 1:** Generalities and demographics of the included studies

**Author, year**	**Journal**	**Study Design**	**Follow-up (*months*)**	**Treatment**	**Procedures (*n*)**	**Female (*%*)**	**Mean age**	**Mean BMI**
Akgun et al. 2015 ^28^	*Arch Orthop Trauma Surg*	Prospective, Randomized	24	Control Group	7	57	32	24.1
				mACI	7	57	32	24.3
Anders et al. 2013 ^64^	*Open Orthop J*	Prospective, Randomized	24	AMIC	8	12	35	27.4
				AMIC	13	23	39	27.7
				Control Group	6	33	41	25.2
Astur et al. 2018 ^65^	*Rev Bras Orthop*	Prospective	12	AMIC	7	14	37	
Bartlett et al. 2005	*J Bone Joint Surg*	Prospective, Randomized	12	Control Group	44	41	34	
				mACI	47		33	
Basad et al. 2010 ^27^	*Knee Surg Sports Traumatol Arthrosc*	Prospective, Randomized	24	mACI	40	38	33	25.3
				Control Group	20	15	38	27.3
Basad et al. 2015 ^15^	*Knee Surg Sports Traumatol Arthrosc*	Prospective	60	mACI	25	37	32	24.0
Becher et al. 2017 ^54^	*J Orthop Surg Res*	Prospective, Randomized	36	mACI	25	32	33	24.9
				mACI	25	16	34	25.6
				mACI	25	40	34	25.1
Behrens et al. 2006 ^25^	*Knee*	Prospective	35	mACI	38	50	35	
Brittberg et al. 2018 ^66^	*Am J Sports Med*	Prospective, Randomized	60	mACI	65	38	35	
				Control Group	63	33	34	
Chung et al. 2014 ^67^	*Knee Surg Sports Traumatol Arthrosc*	Prospective	24	Control Group	12	83	44	
				AMIC	24	42	47	
Cvetanovich et al. 2017 ^68^	*Am J Sports Med*	Prospective	24	Control Group	12	22	17	22.8
				mACI	11	22	17	22.8
				mACI	14	22	17	22.8
De Girolamo et al. 2019 ^47^	*J Clin Med*	Prospective, Randomized	100	AMIC	12	38	30	
				AMIC	12	50	30	
Ebert et al. 2011 ^11^	*Am J Sports Med*	Prospective	60	mACI	44	48	39	25.5
Ebert et al. 2012 ^16^	*Arthroscopy*	Prospective	24	mACI	20	50	34	26.6
Ebert et al. 2015 ^69^	*Am J Sports Med*	Prospective	24	mACI	10	20	39	25.8
				mACI	13	07	36	25.6
				mACI	9	66	38	25.1
				mACI	15	53	37	25.3
Ebert et al. 2017 ^70^	*Am J Sports Med*	Prospective	60	mACI	31	51	35	26
Efe et al. 2011 ^71^	*Am J Sports Med*	Prospective	24	mACI	15	60	26	
Enea et al. 2013 ^72^	*Knee*	Retrospective	22	AMIC	9	45	48	
Enea et al. 2015 ^73^	*Knee*	Retrospective	29	AMIC	9	44	43	
Ferruzzi et al. 2008 ^74^	*J Bone Joint Surg*	Prospective	60	Control Group	48	38	32	
				mACI	50	28	31	
Gille et al. 2013 ^75^	*Arch Orthop Trauma Surg*	Prospective	24	AMIC	57	33	37	
Gobbi et al. 2009 ^76^	*Am J Sports Med*	Prospective	60	mACI	34	32	31	
Gudas et al. 2018 ^77^	*J Orthop Surg*	Retrospective	54	AMIC	15	33	31	
Hoburg et al. 2019 ^33^	*Orthop J Sports Med*	Prospective	63	mACI	29	48	16	21.3
			48	mACI	42	29	27	24.1
Kon el al. 2011 ^61^	*Am J Sports Med*	Prospective	61	Control Group	22	32	46	24.7
			58	mACI	39	35	45	25.6
Lahner et al. 2018 ^78^	*Biomed Res Int*	Prospective	15	AMIC	9		48	29.3
Lopez-Alcorocho et al. 2018 ^79^	*Cartilage*	Prospective	24	mACI	50	30	35	
Macmull et al. 2011 ^80^	*Int Orthop*	Prospective	66	Control Group	24	29	16	
				mACI	7			
Macmull et al. 2012 ^81^	*Am J Sports Med*	Prospective	45	Control Group	25	80	35	
			35	mACI	23	61	35	
Marlovits et al. 2012 ^82^	*Am J Sports Med*	Prospective	60	mACI	24	12	35	
Meyerkort et al. 2014 ^83^	*Knee Surg Sports Traumatol Arthrosc*	Prospective	60	mACI	23		42	
Migliorini et al. 2021 ^84^	*LIFE*	Prospective	43.7	AMIC	52	35	30	27.1
			39.5	Control Group	31	32	31	26.5
Migliorini et al. 2021 ^85^	*LIFE*	Prospective	45.1	AMIC	27	48	36	26.9
			49.1	Control Group	11	55	31	25.1
Nawaz et al. 2014 ^32^	*J Bone Joint Surg*	Retrospective	74	Control Group	827	40	34	
				mACI				
Nejadnik et al. 2010 ^31^	*Am J Sports Med*	Retrospective	24	mACI	36	50	43	
				Control Group	36	44	44	
Niemeyer et al. 2008 ^30^	*Arch Orthop Trauma Surg*	Retrospective	38	Control Group	95		34	25.1
				mACI				
Niemeyer et al. 2016 ^86^	*Am J Sports Med*	Prospective, Randomized	12	mACI	25	33	33	24.9
				mACI	25	16	34	25.6
				mACI	25	40	34	25.1
Niemeyer et al. 2019 ^87^	*Orthop J Sports Med*	Prospective, Randomized	24	mACI	52	36	36	25.7
				Control Group	50	44	37	25.8
Saris et al. 2014 ^88^	*Am J Sports Med*	Prospective, Randomized	24	mACI	72	37	35	26.2
				Control Group	72		33	26.4
Schagemann et al. 2018 ^89^	*Arch Orthop Trauma Surg*	Retrospective	24	AMIC	20	35	38	27.0
				AMIC	30	43	34	23.9
Schiavone Panni et al. 2018 ^90^	*Knee Surg Sports Traumatol Arthrosc*	Retrospective	84	AMIC	21			
Schneider et al. 2011 ^91^	*Am J Sports Med*	Prospective	30	mACI	116	42	33	24.5
Schüttler et al. 2019 ^92^	*Arch Orthop Trauma Surg*	Prospective	60	mACI	23	34		27.8
Siebold et al. 2018 ^93^	*Knee Surg Sports Traumatol Arthrosc*	Prospective	35	mACI	30	36	36	23.8
Steinwachs et al. 2019 ^94^	*Knee*	Retrospective	6	AMIC	93	28	42	
Volz et al. 2017 ^95^	*Int Orthop*	Prospective, Randomized	60	AMIC	17	29	34	27.4
				AMIC	17	11	39	27.6
				Control Group	13	23	40	25.0
Zeifang et al. 2010 ^96^	*Am J Sports Med*	Prospective, Randomized	24	mACI	11	45	29	
				Control Group	10	00	30	

## Discussion

According to the main findings of the present systematic review, AMIC performed better than mACI for chondral defects of the knee at ~40 months follow-up. The rate of complications was noticeably lower in the AMIC group. While the Tegner and VAS scores were similar, the mean difference of the Lysholm and IKDC scales exceeded the minimally clinically important difference (MCID) in favour of the AMIC group.^21^^,^^24^

mACI has been largely performed in patients with focal chondral defects of the knee.^25^^,^^26^ For the mACI procedure, an arthroscopy of the knee is performed first to assess cartilage status, identify the chondral defect and harvest chondrocytes from a non-weightbearing zone of the distal femur.^27–29^ Autologous chondrocytes are subsequently extracted and cultivated, and expanded *in vitro* for ~3 weeks, over a membrane that acts as medium for cell proliferation.^30^^,^^31^ In a second-step surgery, the defect is debrided and the membrane is secured into the defect.^32^^,^^33^ The current literature presents several clinical trials reporting the surgical outcomes of mACI. However, there are still controversies. The optimal surgical approach, whether arthrotomy, mini-arthrotomy or arthroscopy, has not been clarified. Additionally, there are several different membranes used for expansion (resorbable cell-free or cell-based, synthetic), and the most appropriate type of fixation (suture or fibrin glue) is still unclear.^34–39^

**Table 2:** Characteristics of the two cohorts at baseline (n.s.: not significant)

**Endpoint**	**AMIC (*n* = 373)**	**mACI (*n* = 1237)**	***P***
Follow-up (*months*)	37.8 ± 29.9	39.8 ± 17.2	n.s.
Women	34% (125 of 373)	37% (455 of 1237)	n.s.
Mean age	28.2 ± 6.0	33.5 ± 6.5	n.s.
Mean BMI	26.1 ± 1.6	25.9 ± 1.2	n.s.
Right side	33% (124 of 373)	52% (643 of 1237)	n.s.
Defect size (*cm^2^*)	3.5 ± 0.9	3.8 ± 1.0	n.s.
VAS	6.4 ± 0.9	5.6 ± 1.1	n.s.
Tegner	4.0 ± 1.4	3.1 ± 1.3	n.s.
Lysholm	54.1 ± 12.6	53.7 ± 10.7	n.s.
IKDC	47.0 ± 9.1	40.2 ± 8.3	n.s.

**Table 3:** Results of Tegner and IKDC scores (n.s.: not significant)

**Endpoint**	**AMIC**	**mACI**	**MD**	***P***
VAS	2.8 ± 2.2	2.9 ± 1.3	0.07	n.s.
Tegner	4.4 ± 0.6	4.7 ± 0.8	0.3	n.s.
Lysholm	81.9 ± 7.1	65.7 ± 28.2	16.1	0.02
IKDC	79.2 ± 10.4	71.5 ± 6.3	7.7	0.03

**Table 4:** Results of complications (n.s.: not significant)

**Complications**	**AMIC**			**mACI**			**OR**	**95% CI**	***P***
	**events**	**obs**	**rate**	**events**	**obs**	**rate**			
Hypertrophy	0	96	0	29	381	7.6	0.1	0.0 to 1.0	0.05
Failure	2	114	1.8	41	562	7.3	0.2	0.0 to 0.9	0.04
Knee Arthroplasty	2	126	1.6	2	64	3.1	0.5	0.0 to 3.6	n.s.
Revision Surgery	7	117	6.0	39	328	11.9	0.5	0.2 to 1.0	0.07

Recently, AMIC has gained increasing interest.^36^^,^^40–43^ Differently from mACI, which uses laboratory expanded autologous chondrocytes, AMIC is a single session procedure which exploits the regenerative potential of bone marrow derived mesenchymal stem cells (BM-MSCs).^14^^,^^44^ After defect debridement and curettage, microfractures are performed.^45^^,^^46^ A membrane is then placed into the defect. BM-MSCs from the subchondral layer migrate into the membrane and regenerate the hyaline cartilage layer.^12^^,^^47^^,^^48^ Similar to mACI, AMIC can be performed through arthrotomy, mini-arthrotomy or arthroscopy.^49^^,^^50^ However, AMIC is more cost-effective, since it requires only one surgical step, avoiding *in vitro* cell expansion. Moreover, along with the avoidance of chondrocyte harvesting, AMIC should lead to less morbidity and faster recovery. These features make AMIC attractive to both surgeons and patients. We were unable to identify clinical studies which directly compare AMIC versus mACI for chondral defects of the knee: this is the single most important limitation of the available literature. Future studies should establish the most appropriate strategy for knee chondral defects. We hypothesize that the AMIC procedure will promote faster recovery and result in higher patient satisfaction.

We point out that all statistical analyses were performed regardless of the surgical approach. Indeed, authors performed the procedures using arthrotomy, mini-arthrotomy or arthroscopy. The mACI cohort included a larger number of studies and related procedures compared with the AMIC group. This discrepancy may generate biased results and influence the rate of uncommon complications related to poorer outcome. Given the lack of quantitative data, the average return to daily activities and/or sport participation were not investigated. All the membranes considered in the present investigation were cell-free and bioresorbable (collagenic or hyaluronic): this study did not consider cell-based or more innovative synthetic scaffolds.^51–59^ Moreover, the typology of membrane fixation (fibrin glue, suture, both methods or no fixation) was not considered as separate. Given the lack of relevant data, it was not possible to overcome these limitations. Many authors did not differentiate between primary and revision settings, and several studies included patients who received combined surgical procedures. Two studies^60^^,^^61^ performed membrane-assisted autologous chondrocyte transplantation (mACT). In mACT procedures, chondrocytes are harvested, cultivated and expanded into a membrane in the same fashion of mACI. The chondrocyte-loaded membrane is then carefully implanted into the defect using custom-made instruments in a full-arthroscopic fashion.^62^^,^^63^ Given these similarities, we analysed mACT and mACI as a single entity. The lack of detailed information did not allow us to analyse the aetiology of chondral defects as separate data sets. These limitations suggest cautious interpretation of the conclusions of this study.

## Conclusion

AMIC may provide better outcomes than mACI for chondral defects of the knee. Further studies are needed to validate these results in a clinical setting.

## Data Availability

The data underlying this article are available in the article and in its online supplementary material.
